# Effect of polyaspartic acid and different dosages of controlled-release fertilizers on nitrogen uptake, utilization, and yield of maize cultivars

**DOI:** 10.1080/21655979.2020.1865608

**Published:** 2021-02-04

**Authors:** Peng-Tao Ji, Xiang-Ling Li, Yu-Juan Peng, Yue-Chen Zhang, Pei-Jun Tao

**Affiliations:** a College of Agronomy, Hebei Agricultural University, Baoding, Hebei, China; b Hebei Normal University of Science & Technology, Changli, P.R. China

**Keywords:** Polyaspartic acid, controlled-release fertilizer, nitrogen uptake, partial nitrogen productivity, yield

## Abstract

The effects of polyaspartic acid and different controlled-release fertilizers with urea on dry matter accumulation and distribution, nitrogen absorption and accumulation, and the activities of enzymes involved nitrogen metabolism and yield of corn were studied by using xianyu (XY688), a maize nitrogen efficient cultivar, and Jifeng NO.2 (JF2), a maize nitrogen-inefficient cultivar, as experimental materials and through random blocks experimental design in 2019. For XY688, polyaspartic acid chelated nitrogen fertilizer (PASPN) had the highest yield, which was 21.34% higher than N0 treatment. For JF2, it also had the highest yield under PASPN combined urea treatment, which was 23.44% higher than N0 (no nitrogen fertilizer), and JF2 had a 9.7% lower yield under XY688 treatment. For XY688, PASPN treatment had the largest nitrogen uptake in grain, up to 3.14 kg/hm^2^, and PASPN treatment increased 17.4% compared with N0. For JF2, grain nitrogen uptake was also the highest under PASPN treatment, which was significantly different from other treatments. Nitrogen uptake was 3.16 kg/hm^2^, which increased 37.4% compared with N0. Compared with JF2, XY688 showed higher nitrogen uptake efficiency, nitrogen utilization efficiency, and partial nitrogen productivity. For XY688, the highest nitrogen absorption efficiency was SU3 (slow-release urea and ordinary urea) treatment (0.36 kg/kg). The partial nitrogen productivity and harvest index of PASPN treatment were the highest and significantly different from other treatments. The partial nitrogen productivity of PASPN treatment was 57.02 kg/kg. These results can provide help for the further researches of the rational utilization and absorption of nitrogen fertilizer.

## Graphical abstract

**Figure:**
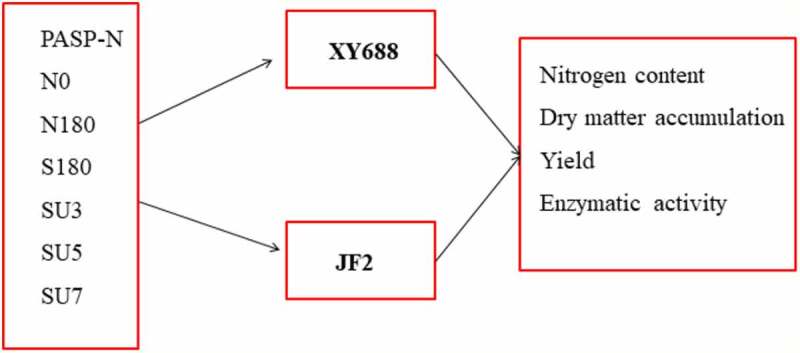

## 1. Introduction

Corn and wheat are not only the two main cash crops in China, but also the main grain crops in the world [1]. Nitrogen fertilizer plays an important role in crop growth and yield [2]. China is the world’s largest consumer and producer of nitrogen fertilizer, accounting for 52% of the world in nitrogen use from 1990 to 2009 [3–5]. However, excessive nitrogen fertilizer is bound to cause a series of problems, not only resulting in the reduction of nitrogen use efficiency and loss of resources, but also brings great pollution to the environments [6], such as soil acidification and water, air pollution [7,8]. Therefore, it is of importance to improve the utilization rate of nitrogen fertilizer and the rational use of chemical fertilizers in agricultural production [9]. To improve the content of nitrogen in corn, many researches have done, such as land cultivation techniques, fertilization time, and fertilizer variety, etc [10,11].

On the other hand, the nitrogen absorption of maize was significantly correlated with varieties with different efficiency. The total nitrogen absorption of high-efficiency cultivars was higher than that of low-efficiency cultivars. Moreover, the nitrogen absorption capacity of high-efficiency cultivars after silking stage was stronger [12]. The nitrogen efficiency, nitrogen absorption efficiency and nitrogen use efficiency of maize with different genotypes were different . Based on the characteristic, maize varieties can be divided into low-efficiency cultivar and high-efficiency cultivar. The essence of both low nitrogen and high nitrogen-efficient cultivar was that the yield of the varieties had a high response to nitrogen supply [13–15]. The difference between the two cultivar was that the low N efficient varieties showed a higher N response in the low N supply condition, while the high N efficient varieties showed a higher N response in the high N supply condition [16,17]. Therefore, they will show different characteristics in the physiological mechanism of high nitrogen nutrition. From the analysis of the relationship between nitrogen and carbon, the accumulation of plant nitrogen is the main factor limiting the crop nitrogen efficiency (yield) under the condition of insufficient N supply. Under the condition of sufficient N supply, the coordination of plant nitrogen metabolism and its relationship become the main factors limiting nitrogen efficiency [15,18].

Compared with traditional nitrogen fertilizer, controlled release fertilizer has been applied more and more recently. Controlled release fertilizer can improve nitrogen use efficiency and further increase crop yield. The fertilizer effect time is long, which will not cause premature aging of corn and reduce environmental pollution [19–21]. Having free carboxyl and amide groups [22], polyaspartic acid has become a popular environmentally friendly polymer and widely used in water-absorbing materials and fertilizer promoters in recent years [23–25]. The polyaspartic acid chelated nitrogen fertilizer is a new fertilizer developed by many companies at present., which is made with the traditional fertilizer combined with a certain amount of polyaspartic acid, which will not cause loss in the reproduction process because it can inhibit nitrification and ammonification [26]. In this way, nitrogen release is delayed and nitrogen loss is reduced to improve nitrogen use efficiency [27]. Therefore, it is of great significance to study the effects of PASP-N on crop yield and nitrogen utilization in maize.

To improve maize yield and nitrogen absorption and utilization rate, in this study, the effects of different proportions of PASP-N on nitrogen accumulation and production efficiency of maize varieties with high and low nitrogen efficiency were studied in Taixing Mountain Plain, Hebei Province. This helps to the further researches of the rational utilization and absorption of nitrogen fertilizer in agricultural research area.

## 2. Materials and methods

### 2.1. Study sites and materials

The experiment was carried out at Xinji Experimental Station of Hebei Agricultural University (43º31′N,124PERIM37º54′N, 115º12′E) in 2019. The average annual temperature is 12.5°C and the average annual precipitation is 488 mm, of which the summer maize season is 382 mm. The soil is loam. The properties of 0–20 cm layer as follows: 17.79 g/kg of organic matter, 1.21 g/kg of total nitrogen, 64.9 mg/kg of alkaline hydrolysable nitrogen, 23.8 mg/kg of available phosphorus and 120.6 mg/kg of available potassium.

### 2.2. Experimental design

The experiment adopted a random block design with three replications at sites. The summer maize varieties were xianyu 688, a nitrogen-efficient cultivar, and Jifeng 2, a nitrogen-inefficient cultivar, respectively. The experiment plot was 8 m long and 3.6 m wide. Field weeding, plant protection, and other management was the same as local field production. The experiments were divided into 8 treatments: (1) PASP-N, a polyaspartic acid chelating nitrogen fertilizer with a nitrogen content of 180 kg/ha; (2) no nitrogen fertilizer, N0; (3) farmers’ fertilizer practice of N 240 kg·ha^−1^, N240; (4) optimizes N management with 180 kg/ha, N180; (5) slow-release urea 180 kg/ha, S180; (6) slow-release urea (S) and ordinary urea (U) were mixed at 3:7 (SU3), and the nitrogen application rate was 180 kg/hm; (7) the slow-release urea (S) and the ordinary urea (U) were mixed at 5:5 (SU5), and the nitrogen application rate was 180 kg/hm; (8) slow-release urea (S) and ordinary urea (U) were mixed at 7:3 (SU7), and the nitrogen application amount was 180 kg/hm. All fertilizers are applied as base fertilizer. PASPN (polyaspartic acid chelated nitrogen fertilizer) is produced by Hubei Jintaineng Fertilizer Co., LTD., and the contents of N, P_2_O_5_ and K_2_O are 22%, 9%, and 9%, respectively. The fertilization was adopted high polymer net capture technology, which has a high loss control rate and continuously provides nutrients for crops. Slow-release urea is produced by Henan Xinlianxin Fertilizer Co., LTD., and its N, P_2_O^5^ and K_2_O contents are 43%, 5%, and 5%, respectively. The amount of phosphorus and potassium fertilizer applied in each treatment is same, with P_2_O_5_ 90 kg/ha and K_2_O 90 kg/ha, respectively.

### 2.3. Sampling and measurements

#### 2.3.1. Yields and dry matter accumulation

After maize mature, 10 m^2^ was selected in the middle of the plot to measure yield, and representative maize at V6 stage, V12 stage, silking stage, milk stage, dough stage, and physiological maturity stage were taken to measure dry matter, respectively. The activities of glutamate synthetase (GOGAT), glutaminase (GLNS), and glutamic oxalacetic transaminase (GOT) and nitrate reductase (NR) were determined. Three representative plants were selected at the flowering and silking stage and the harvest stage and separated according to their parts (leaves, stems and grains. After being killed for 30 min at 105 °C, fresh samples were dried to constant weight at 85 °C, and weighed and crushed to determine the nutrient content of different organs.

#### 2.3.2. Nitrogen content and nitrogen use efficiency analysis

Analysis of plant samples: after the samples were killed and dried, the stem were cut into small sections, and ground up with the seeds, and digested with H_2_SO_4_-H_2_O_2_ method to determine the total nitrogen content. Nitrogen calculation index formula is as follows:

$$Grain\;N\;uptake\left(kg.hm^{-2}\right)=Grain\;N\;conten\left(g.kg^{-1}\right)\times Grain\;yield\left(kg.hm^{-2}\right)/1000$$

$$Stem\;N\;uptake\left(kg.hm^{-2}\right)=Stem\;N\;conten\left(g.kg^{-1}\right)\times Stem\;dry\;matter\left(kg.hm^{-2}\right)/1000$$

$$Aboveground\;N\;uptake\left(kg.hm^{-2}\right)=Aboveground\;N\;content\left(g.kg^{-1}\right)\times aboveground\;biomass\left(kg.hm^{-2}\right)/1000\;$$

N use effiency%=N output/N input

$$Nitrogen\;harvest\;index\left(NHI\%\right)\;=grain\;Ncontent/plant\;Ncontentatmaturity\times \;100$$

$$Partial\;factor\;productivity\;\left(kg/kg\right)\;=\;grain\;yield\;\left(kg/hm^{2}\right)/N\;input$$

$$Partial\;factor\;productivity(kg/kg)=Grain\;yield(kg.hm^{\left(-2\right)})/N\;input(kg.hm^{\left(-2\right)})$$

### 2.4. Statistical analysis

Microsoft Excel 2013 software was used to sort the data. SPSS 18.0 software was used to analyze the data statistically. One-way ANOVA and LSD methods were used for analysis of variance and multiple comparisons (α = 0.05), and R software was used for correlation analysis.

## 3. Results

Our purpose is to study the different fertilizer treatment on the production and nitrogen accumulation and distribution of dry matter, nitrogen absorption and accumulation of different maize varieties, and whether enzyme activity is closely related to the nitrogen metabolism, enzyme activity and yield. We suppose that the utilization and absorption and utilization rate of fertilizer are related to maize varieties, and the enzyme activity and uptake and conversion is associated with the growth period of maize.

### 3.1. Yield and dry matter accumulation

**Figure 1.**
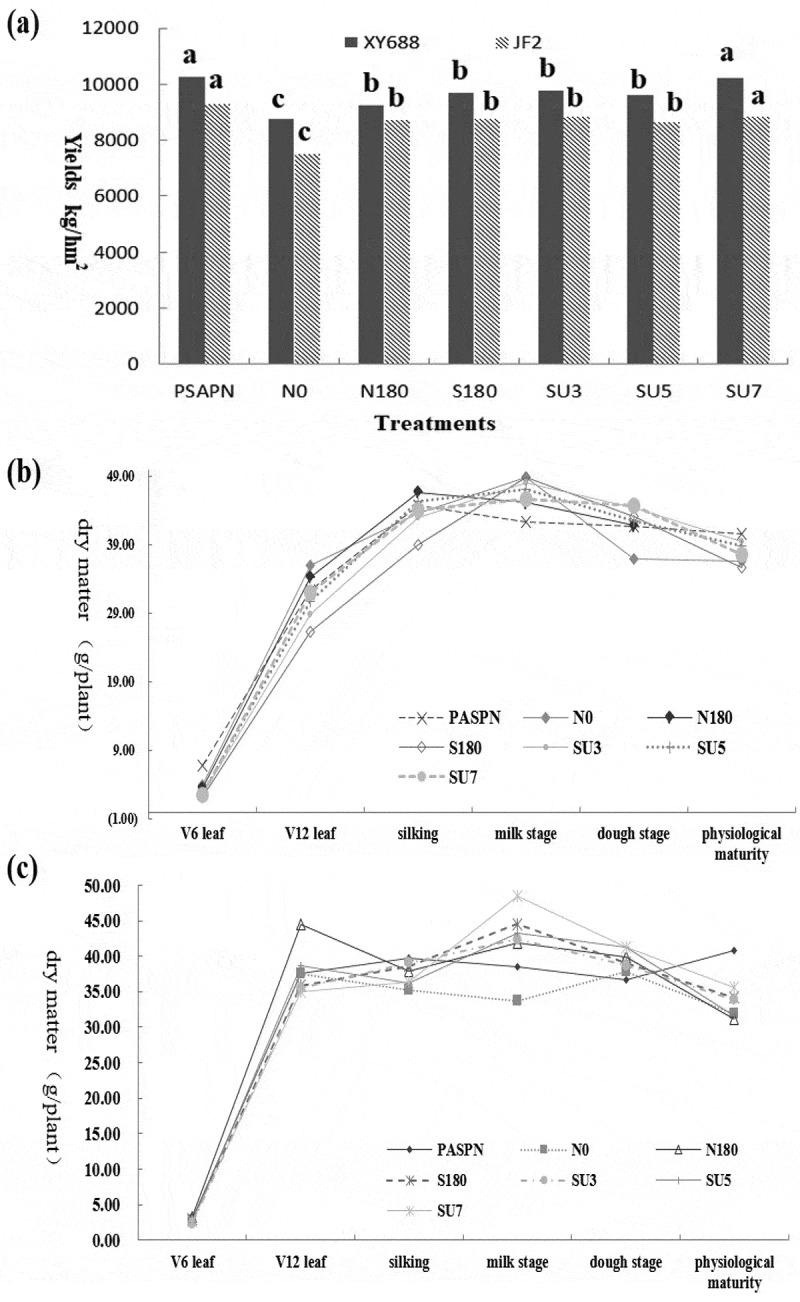
Yield of XY688 and JF2 under different fertilization ratio (a); Variation trend of dry matter of XY688 at different growing stages (b) and Variation trend of dry matter of JF2 at different growing stages (c)

The yield of different fertilization treatments is shown in Figure 1. As shown in the figure, for XY688, it can be seen that the yield of PASPN treatment is the highest under the same nitrogen application amount, which is 21.34% higher than that of N0 treatment, followed by SU7. The yield of controlled-release fertilizer combined with urea treatment was significantly different from N0, and the yield can be increased to a certain extent compared with urea. For JF2, the yield of PSAP combined with urea treatment was the highest, which increased by 23.44% compared with CK treatment, and the yield was significantly lower than XY688. For PSAPN treatment, the yield of JF2 decreased by 9.7% compared with XY688. Single application of urea or single application of controlled-release fertilizer is not as effective as the mixing of the two in terms of yield.

The dry matter accumulation of different treatments in different periods is shown in Figure 1. As for XY688, it can be seen that dry matter basically reached the peak in the milk stage, and then gradually decreased. With the increase of JF2, dry matter peaked at V12 stage, then declined. After the silking stage, XY688 had an increase trend in dry matter compared with JF2. For XY688, the variation of each treatment is not significant, and the trend is basically same, all increased at the beginning and then decreased. For JF2, SU7 treatment had a significant difference in dry matter. In the milk stage, SU7 treatment increased by 25.76% compared with N0 treatment. In the silking stage, the dry matter of N180 treatment was the largest, which was 18.43% higher than N0.

As can be seen from Table 1, maize varieties with different nitrogen efficiency had great differences in dry matter accumulation and ratio at different growing stages, but there was little difference in the effect of nitrogen fertilizer on the two varieties. The largest dry matter accumulation of the two varieties was in silking-dough stage, XY688 and JF2 reached 46% and 52.21%, respectively. In each growth stage, XY688 showed higher biomass accumulation than JF2, except from silking-dough stage, sowing-jointing, jointing-silking stage, dough-maturity stage, 25%, 8.4% and 5.2% higher, respectively. There was a little difference between the two cultivars in the early sowing stage, but the difference began to be obvious from the silking stage to the jointing stage. The dry matter accumulation difference between nitrogen-efficient cultivars and nitrogen-inefficient cultivars mainly appeared at jointing-silking stage and silking-dough stage. Nitrogen-efficient cultivars could maintain high dry matter accumulation rate at late growth stage, which ensured the transportation of carbohydrates to grains at late growth stage.

**Table 1.** Dry matter accumulation and ratio of maize in different growth stages

Treatments		Sowing to jointing		Jointing to silking		Silking to dough		Dough to maturity	
		DMA(g/plant)	RTDA%	DMA(g/plant)	RTDA%	DMA(g/plant)	RTDA%	DMA(g/plant)	RTDA%
XY688	PASPN	9.95 ± 3.48 a	2.81	140.02 ± 6.22 a	42.40	143.09 ± 6.1 b	40.45	60.6 ± 7.5 a	17.15
	N0	5.71 ± 1.24 b	1.79	131.14 ± 15.72 b	42.95	140.8 ± 3.1 b	44.19	40.94 ± 1.3 d	12.85
	N180	5.69 ± 0.39 b	1.68	140.94 ± 9.14 a	43.36	147 ± 3.6 b	43.47	44.56 ± 7 c	13.18
	S180	3.27 ± 0.56 c	0.98	134.83 ± 8.73 b	41.15	154.4 ± 1.3 a	46.00	43.11 ± 8.6 c	12.85
	SU3	4.04 ± 0.82 c	1.17	133.24 ± 7.04 b	39.73	157.44 ± 1.8 a	45.56	50.8 ± 9.28 b	14.72
	SU5	3.95 ± 0.45 c	1.16	141.31 ± 14.17 a	42.60	145.36 ± 2.6 b	42.62	50.4 ± 1.1 b	14.78
	SU7	3.8 ± 0.25	1.10	140.88 ± 6.94 a	41.76	150.86 ± 3.7 a	39.84	50.92 ± 1.9 b	14.70
Average	5.20		1.53	137.48	42	148.42	43.16	48.76	14.32
JF2	PASPN	4.56 ± 1.26 a	1.36	142.92 ± 3.84 a	44.01	133.49 ± 7.9 c	44.58	54.12 ± 9.8 a	16.15
	N0	4.07 ± 1.29 b	1.30	132.23 ± 5.38 b	43.52	139.64 ± 9.6 c	45.57	44.58 ± 1.0 c	11.90
	N180	4.84 ± 1.09 a	1.47	125.07 ± 4 b	39.47	151.17 ± 5.9 b	46.62	48.05 ± 1.7 c	14.60
	S180	4.23 ± 0.17 b	1.29	128.77 ± 3.83 b	40.66	152.5 ± 2.2 b	46.75	41.64 ± 1.3 c	12.73
	SU3	3.76 ± 0.64 b	1.15	124.77 ± 3.4 b	39.33	152.79 ± 9 b	52.21	45.5 ± 9.8 b	13.92
	SU5	3.69 ± 0.16 b	1.14	115.06 ± 3.09 c	36.73	168.78 ± 5.1 a	47.79	35.75 ± 9.5 d	11.06
	SU7	3.97 ± 0.38 b	1.16	119.21 ± 3.74 c	36.13	162.93 ± 4.8 a	47.81	54.84 ± 1.4 a	16.09
Average	4.16		1.27	126.85	39.98	151.61	47.33	46.35	13.78

Different lowercase letters mean significant difference at 0.05 level, while values with the same letter mean insignificant difference.

For XY688, it can be seen that the dry matter accumulation of PASP chelated urea treatment is the highest at the different growth stages, and significantly different from N0 treatment. The dry matter accumulation reached 9.95 g/plant, 140.02 g/plant, 143.09 g/plant and 60.6 g/plant, at sowing-jointing stage, jointing-silking stage, silking-dough stage, dough-maturity stage, respectively. At the same time, it also can be seen that the dry matter accumulation of controlled-release fertilizer combined with urea showed advantages, and controlled-release fertilizer delayed the release of nitrogen at the middle sowing stage, that is silking-dough stage. Therefore, in addition to its role at the middle sowing stage, the influence of PASPN was still the biggest at the mature stage. SU7 treatment was followed, with dry matter accumulation of 3.8g/plant, 140.88 g/plant, 150.86 g/plant and 50.92 g/plant during the whole growth period. For JF2, the dry matter accumulation of SU5 treatment reached 168.78 g/plant at the silking-dough stage, while the dry matter accumulation of PASPN treatment was the highest at the other growth stages and was significantly different from N0 treatment. The dry matter were 4.56 g/plant, 142.92 g/plant and 54.12 g/plant at sowing-jointing stage, jointing-silking stage, and dough-maturity stage, respectively.

### 3.2. Nitrogen accumulation and distribution

The nitrogen content in stems, leaves, and grains of maize at silking and maturing stages is shown in Table 2. Comparing the two varieties, the overall trend was that the content of XY688 was higher than JF2 in different parts of maize. For XY688, it can be seen that, N180 and SU5 had the highest nitrogen content in the silking stage, which were 1.2 mg/g and 1.18 mg/g, respectively, which increased by 21% and 19% compared with N0. For stem, SU3 had the highest nitrogen content, which was 2.57 mg/g. At the maturity stage, nitrogen content in leaves and stems gradually decreased, mainly concentrated in grains. PASPN had the highest nitrogen content in grains, 3.14 mg/g, which was significantly different from other treatments except SU3 treatment, and the nitrogen content increased by 14.6% compared with N0. Similarly, for JF2, PASPN had the highest nitrogen content in the grains at maturity, which was 3.16 mg/g, an increase of 36.8 mg/g compared with N0. For total nitrogen content, PASPN also the best, at 4.05 mg/g.

**Table 2.** Nitrogen content in different parts of maize at silking stage and ripening stage

Cultivar	Treatment	N content at silking (mg/g)			N content at maturity (mg/g)			
		Leaf	Stem	Total	Leaf	Stem	Grain	Total
XY688	PASPN	1.12 ab	1.99 b	3.11 ab	0.48 b	0.42 b	3.14 a	4.05 a
	N0	0.99 b	1.50 c	2.50 b	0.45 b	0.45 b	2.47 c	3.45 c
	N180	1.20 a	1.85 b	3.04 ab	0.61 a	0.43 b	3.00 b	4.18 a
	S180	1.03 ab	1.99 b	3.03 ab	0.53 ab	0.39 b	2.99 b	4.02 b
	SU3	0.85 b	2.57 a	3.42 a	0.49 b	0.76 b	3.03 a	4.31 a
	SU5	1.18 a	2.16 b	3.34 a	0.47 b	0.81 a	2.76 b	4.08 a
	SU7	0.82 b	2.07 b	2.89 b	0.35 c	0.56 b	2.97 b	3.96 b
JF2	PASPN	0.66 b	1.70 b	2.36 b	0.37 b	0.48 a	3.16 a	4.05 a
	N0	0.96 a	1.60 b	2.56 b	0.53 a	0.51 a	2.31 c	3.37 b
	N180	0.98 a	1.80 b	2.78 a	0.47 b	0.42 a	2.92 ab	3.98 a
	S180	0.78 a	2.06 a	2.84 a	0.56 a	0.46 a	2.81 b	3.98 a
	SU3	0.79 b	1.96 a	2.75 a	0.44 b	0.47 a	2.83 b	3.91 a
	SU5	0.80 b	2.06 a	2.86 a	0.54 a	0.31 b	2.79 b	3.78 a
	SU7	0.94 ab	1.65 b	2.59 b	0.55 a	0.44 a	2.87 b	4.00 a

Different lowercase letters mean significant difference at 0.05 level, while values with the same letter mean insignificant difference.

The nitrogen accumulation in different organs of maize in different growth stages is shown in Table 3. It can be seen that the nitrogen accumulation of JF2 was slightly higher than XY688 at milk stage, but at the maturity stage, XY688 was significantly higher than JF2, indicating that the nitrogen accumulation of high-efficiency nitrogen varieties was relatively high at the late growth stage, while the nitrogen accumulation of low-efficiency nitrogen varieties was mainly concentrated in the early growth stage. At the milk stage, nitrogen is mainly concentrated in the stem, while the content of leaf and ear is less. At the stage of maturity, nitrogen is mainly concentrated in the grains. It can be seen that, XY688 and JF2 had the highest nitrogen accumulation at the maturity stage was PASPN treatment, which was significantly different from other treatments. XY688 had a nitrogen accumulation of 3.138 g/plant, 25.4% higher than N0, followed by SU3 (3.506 g/plant). For JF2, the nitrogen accumulation in PASPN treatment was 3.002 g/plant, 27.7% higher than N0 and significantly different from other treatments. SU7 was followed by nitrogen accumulation of 2.855 g/plant.

**Table 3.** Nitrogen accumulation in different organs of maize at Suckling stage and complete stage

		Milk stage				Maturity stage			
Cultivar	Treatments	Leaf (g/plant)	Stem (g/plant)	Ear (g/plant)	Grain (g/plant)	Leaf (g/plant)	Stem (g/plant)	Ear (g/plant)	Grain (g/plant)
688	PASPN	0.173 a	1.164 a	0.150 bc	0.311 b	0.010 d	0.447 c	0.012 d	3.138 a
	N0	0.114 b	1.104 a	0.212 a	0.243 c	0.027 c	0.450 c	0.047 c	2.501 c
	N180	0.084 c	0.983 b	0.239 a	0.418 a	0.071 a	0.421 c	0.068 bc	2.982 c
	S180	0.066 c	0.838 b	0.130 c	0.334 b	0.048 b	0.392 c	0.066 bc	3.009 b
	SU3	0.074 c	1.062 a	0.152 bc	0.423 a	0.009 d	0.734 ab	0.007 d	3.016 b
	SU5	0.029 c	0.965 b	0.060 d	0.307 b	0.008 d	0.828 ab	0.046 c	2.793 c
	SU7	0.059 c	1.406 a	0.166 bc	0.331 b	0.018 d	0.551 b	0.071 a	3.005 b
JF2	PASPN	0.019 b	1.198 c	0.086 b	0.360 b	0.035 d	0.487 b	0.012 d	3.002 a
	N0	0.023 a	1.144 c	0.020 c	0.250 c	0.022 d	0.502 a	0.008 d	2.351 c
	N180	0.011 b	1.295 b	0.012 c	0.375 b	0.066 bc	0.402 c	0.106 d	2.830 b
	S180	0.019 b	1.287 b	0.002 d	0.409 a	0.054 c	0.449 c	0.102 d	2.690 b
	SU3	0.007 c	1.255 bc	0.065 b	0.288 c	0.089 a	0.478 b	0.095 a	2.723 b
	SU5	0.019 b	1.322 a	0.077 b	0.302 bc	0.058 c	0.326 d	0.082 b	2.727 b
	SU7	0.021 a	0.900 d	0.121 a	0.320 b	0.076 b	0.453 c	0.077 c	2.855 b

Different lowercase letters mean significant difference at 0.05 level, while values with the same letter mean insignificant difference.

### 3.3. Effects of different fertilization treatments on nitrogen uptake and use efficiency

The effects of different fertilizer ratios on the nitrogen uptake of grains and plants were significantly different (Table 4). For XY688, PASPN had the highest nitrogen uptake in grain, up to 3.14 kg/hm^2^, which was significantly different from other treatments except for the SU3 treatment. N0 had the lowest nitrogen uptake, and PASPN increased by 26.6% compared with N0. For plant nitrogen uptake, PASPN treatment increased by 17.4% compared with N0. While for JF2, the nitrogen uptake of grain was also the highest under PASPN, which was significantly different from other treatments. The nitrogen uptake was 3.16 kg/hm^2^, which was 37.4% higher than N0 treatment, and the nitrogen uptake of plants was also significantly different compared with N0 treatment.

**Table 4.** Effects of different fertilization treatments on Nitrogen uptake and yield

Cultivar	Treatment	Grain N uptake kg/hm^2^	Plant N uptake kg/hm^2^
XY688	PASPN	3.14 a	4.05 b
	N0	2.48 c	3.45 c
	N180	3 b	4.19 ab
	S180	2.98 b	4.03 b
	SU3	3.03 ab	4.31 a
	SU5	2.76 b	4.08 ab
	SU7	2.97 b	3.96 b
JF2	PASPN	3.16 a	4.05 a
	N0	2.3 c	3.37 b
	N180	2.92 b	3.98 a
	S180	2.81 b	3.97 a
	SU3	2.83 b	3.91 a
	SU5	2.79 b	3.78 a
	SU7	2.87 b	3.89 a

The effects of different fertilizer ratios on nitrogen use efficiency are shown in Table 5. In general, XY688 had higher nitrogen absorption efficiency, nitrogen use efficiency, and partial nitrogen productivity than JF2, because XY688 is a high nitrogen absorption efficiency cultivar. For XY688, the highest nitrogen absorption efficiency was SU3, 0.36 kg/kg. NUE was different between each treatment, for SU7, NUE reached 44.95%, and increased by 13.5% compared with N0, and nitrogen partial productivity and harvest index PASPN was the highest, and significant difference with other treatments. Compared with N0, S180, SU3, SU5, and SU7, the partial nitrogen productivity of PASPN was 57.02 kg/kg, which increased by 14.2%, 5.9%, 5%, 6.8% and 0.3%, respectively. For JF2, there was no significant difference in nitrogen uptake efficiency and nitrogen use efficiency except for N0. The maximum treatment of partial productivity of nitrogen fertilizer was PASPN, up to 51.47 kg/kg, which was significantly different from other treatments and 9.3% higher than N0. The PASPN treatment of nitrogen harvest index was 0.78%, which was significantly different from other treatments. Compared with JF2, partial nitrogen productivity of XY688 increased by 10.78%.

**Table 5.** Effects of different fertilization treatments on nitrogen utilization and harvest index

Cultivar	Treatment	Nitrogen absorption efficiency Kg/kg	Nitrogen efficiency %	Partial factor productivity Kg/kg	Nitrogen harvest index %
XY688	PASPN	0.34 b	42.84 a	57.02 a	0.78 a
	N0	0.29 c	39.59 a	49.89 c	0.72 b
	N180	0.35 ab	39.76 a	51.30 c	0.72 b
	S180	0.34 b	40.91 a	53.80 bc	0.74 b
	SU3	0.36 a	40.70 a	54.28 b	0.70 b
	SU5	0.34 ab	40.19 a	53.37 bc	0.68 b
	SU7	0.33 b	44.95 a	56.83 ab	0.75 b
JF2	PASPN	0.338 a	41.57 a	51.47 a	0.78 a
	N0	0.281 b	37.49 b	47.10 c	0.69 c
	N180	0.332 a	39.90 a	48.42 b	0.73 b
	S180	0.331 a	40.57 a	48.75 b	0.71 bc
	SU3	0.326 a	40.79 a	49.17 b	0.72 bc
	SU5	0.315 a	41.85 a	47.86 b	0.74 b
	SU7	0.333 a	40.76 a	48.99 b	0.72 bc

### 3.4. The physiological activity of ear leaf

#### 3.4.1. Nitrate reductase (NR)

**Figure 2.**
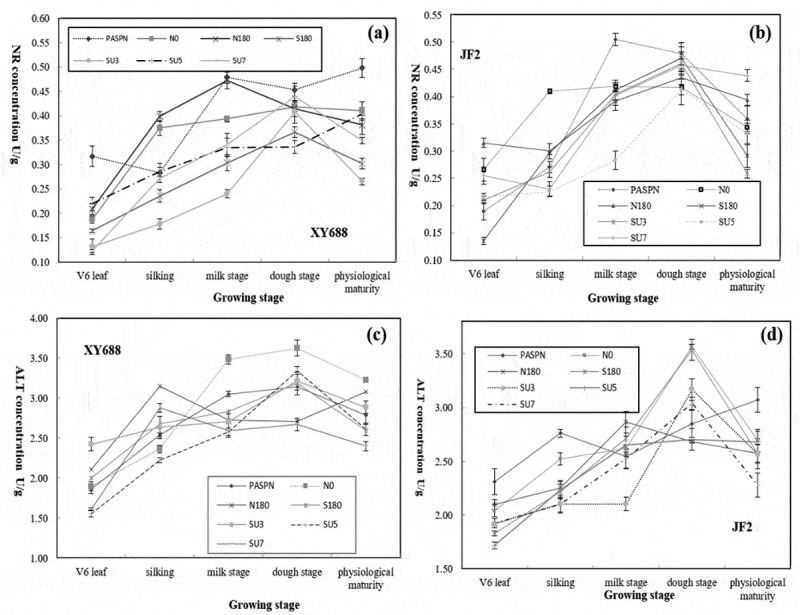
Effects of different fertilization ratio on nitrate reductase concentration in Maize (a and b) and Effect of different fertilization ratio on the concentration of ALT in Maize (c and d)

The nitrate reductase activity of ear leaves under different fertilization ratios is shown in Figure 2. As for XY688 (Figure 2a), PASPN treatment showed a decrease at first and then a increasing trend before the milk stage. At the milk stage, PASPN treatment is 21.9%, 16%, 58%, 100.4%, 43.2% and 40.7% higher than N0, N180, S180, SU3, SU5, and SU7, respectively. At maturity, PASPN was significantly higher than other treatments, with a content of 0.5 U/g. For the nitrogen-inefficient JF2 (Figure 2b), except for the PSAPN, other treatments reached the peak at the waxes ripening stage, the PASPN treatment had the highest concentration of 0.48 U/g at the dough stage, and SU7 had the maximum concentration of 0.44 U/g at the mature stage.

#### 3.4.2. Aspartate aminotransferase(ALT)

**Figure 3.**
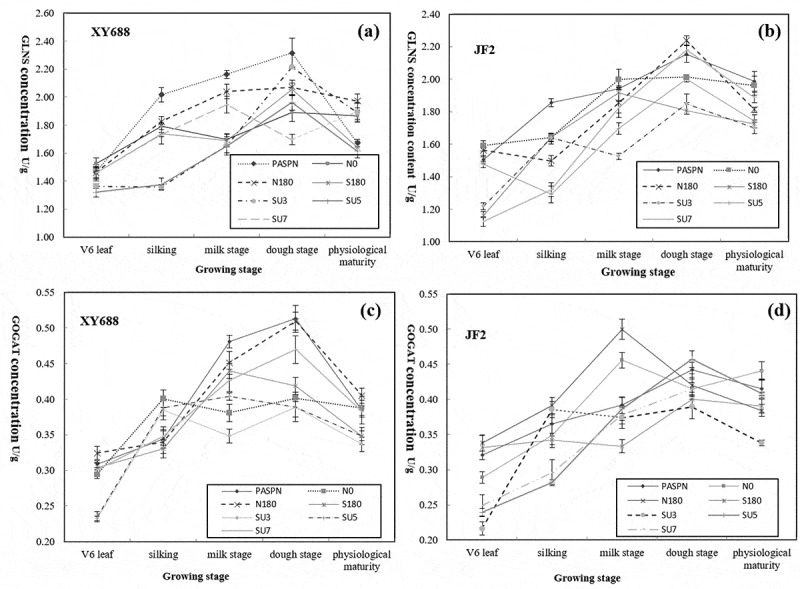
Effect of different fertilization ratio on the concentration of ALT in Maize (a and b); and Effects of different fertilization ratio on GLNS concentration in Maize (c and d)

ALT activity in ear leaves fluctuated with the growth period under different fertilization ratios (Figure 3). For JF2 (Figure 3b), and it can be seen that the ALT content in N0, S180, SU3, and SU7 treatment was in the peak state at the dough stage, which is 3.57 U/g, 3.54 U/g, 3.17 U/g and 3.04 U/g, respectively. However, at the maturity stage, the ALT content of PASPN was still the highest with a value of 3.07 U/g, which was 13.8%, 19.2%, 19.4%, 19.4%, 14.4% and 34.6% higher than N0, N180, S180, SU3, SU5, and SU7, respectively. For XY688 (Figure 2), N0 treatment showed a good advantage at the late growth stage, reaching the maximum value of 3.62 U/g at the maturity stage.

#### 3.4.3. Glutamine synthetase (GLNS)

For XY688 (Figure 3), the increasing trend of GLNS SU3 and SU5 at the silking stage was slower than other treatments, and the decreasing trend of S180 and N0 at the milking stage and then showed increase trend, while at the dough stage, the activity of GLNS in PASPN treatment reached the maximum at 2.31 U/g. However, at the maturity stage, the maximum GLNS activity of N180 treatment reached 1.97 U/g. For JF2 (Figure 3b), PASPN reached its maximum value at 1.99 U/g at the maturity stage, which was 1.5%, 9.8%, 15.37%, 17.15%, 14.3%, and 5.4% higher than N0, N180, S180, SU3, SU5, and SU7, respectively.

#### 3.4.4. Glutamate synthase (GOGAT)

For XY688 (Figure 3), the activities of glutamate synthase increased linearly from the silking stage to the maturity stage in PASPN and N180 treatments, and reached the peak value at the dough stage, 0.51 U/g. For JF2 (Figure 3b), the GOGAT in N180 and N0 peaked 0.5 U/g and 0.46 U/g, respectively, and subsequently decreased, while in other treatments peaked at dough stage and subsequently decreased. At the maturity stage, the maximum value of GOGAT treated with N0 and SU7 was 0.44 U/g.

## 4. Discussion

### 4.1. Effect of PASP and controlled-release fertilizer on yield increase of maize

PASP, because of the molecular chain carries free carboxyl and amide groups, it has a strong adsorption capacity for ions, so it can reduce the loss of nutrients and increase the nutrient content in the soil [26]. Therefore, the root system can obtain abundant nutrients and effectively absorb and utilize nutrients [28]. The application of PASP or PASP-N to maize and mustard could significantly improve nutrient absorption, growth rate, biomass, grain yield, and nutrient use efficiency [26,29]. In this study, the yield of PASPN treatment was the highest, which was 21.34% higher than N0. The yield of controlled-release fertilizer combined with urea treatment was significantly different with N0, and the yield could be increased to a certain extent compared with that of urea application alone. JF2 increased by 23.44% compared with N0, and its yield was significantly lower than XY688. For PSAPN treatment, the yield of JF2 decreased by 9.7% compared with XY688.

### 4.2 Effects of different fertilizer ratio on nitrogen accumulation and use efficiency

Nitrogen production efficiency is evaluated as one of the main indexes of N element management. Controlling the accumulation of dry matter and nitrogen and distribution of nitrogen is an effective way to improve the use efficiency of nitrogen without affecting the crop yield [30,31]. Previous studies have shown that increasing the demand and supply of nitrogen is an effective way to improve crop yield and nitrogen use efficiency [27,32]. Therefore, reasonable nitrogen application is very important to improve crop yield and NUE. PASP is widely recognized as a substance that promotes crop growth, and increases crop yield and nitrogen efficiency [45,29,33]. In this experiment, it can be seen that PASPN can significantly increase maize yield and improve nitrogen utilization to a certain extent. Previous studies concluded that, under the same farming methods and varieties, the application of controlled-release fertilizer combined with urea had an obvious improvement in N absorption compared with the traditional application of urea [34].

This may be because the controlled-release fertilizer is better suited to corn nitrogen requirements. However, the traditional urea capacity leads to excess nitrogen at the early growth stage and lack of nitrogen at the late growth stage, and also leads to the leaching of nitrate [35]. Controlled-release fertilizer can solve this problem, reducing leaching and increasing nitrogen use efficiency of corn at the whole growth period [36]. In this study, the nitrogen accumulation of high-efficiency cultivar was relatively large at the late growth stage, while the nitrogen accumulation of low-efficiency cultivars was mainly at the early growth stage. At the milk stage, nitrogen is mainly concentrated in the stem, while the content of leaf and ear is less. At the stage of ripening, nitrogen is mainly concentrated in the grain. At the maturity stage, for XY688 and JF2, the highest nitrogen accumulation in grain was PASPN, which was significantly different from other treatments. Because XY688 has high nitrogen absorption, XY688 has higher nitrogen absorption efficiency, nitrogen use efficiency, and nitrogen partial productivity than JF2. For XY688, the highest nitrogen absorption efficiency is SU3 treatment, 0.36 kg/kg. Compared with N0, S180, SU3, SU5, and SU7, the partial nitrogen productivity of PASPN treatment was 57.02 kg/kg, which increased by 14.2%, 5.9%, 5%, 6.8%, and 0.3%, respectively. For JF2, the maximum treatment of partial productivity of nitrogen fertilizer was PASPN treatment, which was 51.47 kg/kg, which was significantly different from other treatments and 9.3% higher than N0, and the PASPN treatment of nitrogen harvest index was 0.78%, which was significantly different from other treatments. Compared with JF2, partial nitrogen productivity of XY688 increased by 10.78%. Therefore, it can be seen that single urea application has no significant improvement in nitrogen uptake and nitrogen use efficiency, while PASP and different controlled-release fertilizers and urea ratios can more effectively improve nitrogen productivity and use efficiency.

### 4.3. Regulatory effects of different fertilization ratios on key enzymes of maize nitrogen metabolism

Studies have found that PASP combined with conventional fertilizer can improve nitrate reductase activity in maize seedling leaves. NR is a rate-limiting enzyme in nitrogen metabolism and is affected by temperature, light and CO_2_ concentration. Glutamine synthetase is a key enzyme for ammonia assimilation in plants, catalyzing the formation of glutamine by NH_3_ and glutamic acid in plants [37]. Studies have shown that exogenous nitrate would have a positive correlation with NR under the condition of low nitrogen. NR and surfactant change dynamics are related to crop growth characteristics and crop demand [38]. The activity level reflects the growth and development of crops, which has a great influence on crop yield and quality [39]. In this study, the concentration of NR in the nitrogen-efficient cultivars tended to peak at the dough stage, while the concentration of NR in the nitrogen-inefficient cultivars tended to decrease at the dough stage and always increased before the dough stage. However, the response of different fertilization ratios to NR was not obvious, which maybe because it was tested for only one year.

Glutamine synthase has two activities: invertase and synthase. The increase of activity was beneficial to ammonium assimilation and nitrogen transport [40]. The improvement of GS activity can drive the nitrogen metabolism to strengthen, and promote the synthesis and transformation of amino acids. The effects of exogenous nitrogen on nitrogen anabase expression in higher plants vary with plant species, tissues, organs, environment, and nitrogen sources. Glutamine synthetase and alanine aminotransferase had synergistic effect on NR [41,42]. GOT and GLNS are two important amino transferases in plants, which catalyze the reversible transfer of amino acids from glutamic acid to oxalic acid and pyruvate, respectively, [43–45]. In this study, JF2, GLNS activity reached its maximum at maturity, which was higher than N0, N180, S180, SU3, SU5 and SU7. XY688, for the activity of GOGAT, PASPN and N180 increased linearly from the silking stage to the maturity stage, and reached the peak value at the dough stage.

## 5. Conclusions

The effects of polyaspartic acid and different controlled-release fertilizers with urea on dry matter accumulation and distribution, nitrogen absorption and accumulation, and the activities of enzymes involved nitrogen metabolism and yield of maize were studied by using xianyu (XY688), a maize nitrogen-efficient cultivar, and Jifeng NO.2 (JF2), a maize nitrogen-inefficient cultivar, as experimental materials and through random blocks experimental design in 2019. We found that PASP chelated urea significantly increased the yield of maize by 21.34–23.44% compared with CK treatment, and controlled-release fertilizer combined with urea application can significantly increase the yield of maize compared with urea application alone, and the optimal ratio is SU7. Additionally, it was found that both PASP and controlled-release fertilizer could improve the nitrogen absorption and nitrogen use efficiency of crops, and the nitrogen-efficient cultivars had better performance than the nitrogen-inefficient cultivars under the same nitrogen application level. These results will provide data and theoretical basis for improving crop yield, quality, and guiding the use of nitrogen fertilizer in the future.

## Highlights

1. The combination of all the treatments to analysis the difference in N, dry matter and yield;
2. This experiment showed the effect on different enzymes
3. Nitrogen efficient cultivar and nitrogen inefficient cultivar were both conclude in this article.
4. Polyaspartic acid chelated urea significantly increased the yield of maize by 21.34-23.44% compared with CK treatment;
5. The nitrogen efficient cultivars had better performance than the nitrogen inefficient cultivars under the same nitrogen application level.
